# Sensitive determination of hydrazine using poly(phenolphthalein), Au nanoparticles and multiwalled carbon nanotubes modified glassy carbon electrode

**DOI:** 10.3906/kim-2009-12

**Published:** 2021-02-17

**Authors:** Müge HATİP, Süleyman KOÇAK, Zekerya DURSUN

**Affiliations:** 1 Department of Chemistry, Faculty of Science and Letters, Manisa Celal Bayar University, Manisa Turkey; 2 Applied Science Research Center (ASRC), Manisa Celal Bayar University, Manisa Turkey; 3 Department of Chemistry, Faculty of Science, Ege University, İzmir Turkey

**Keywords:** Gold nanoparticles, poly(phenolphthalein), multiwalled carbon nanotube, hydrazine, voltammetry, amperometry

## Abstract

This study reports a detailed analysis of an electrode material containing poly(phenolphthalein), carbon nanotubes and gold nanoparticles which shows superior catalytic effect towards to hydrazine oxidation in Britton–Robinson buffer (pH 10.0). Glassy carbon electrode was modified by electropolymerization of phenolphthalein (PP) monomer (poly(PP)/GCE) and the multiwalled carbon nanotubes (MWCNTs) was dropped on the surface. This modified surface was electrodeposited with gold nanoparticles (AuNPs/CNT/poly(PP)/GCE). The fabricated electrode was analysed the determination of hydrazine using cyclic voltammetry, linear sweep voltammetry and amperometry. The peak potential of hydrazine oxidation on bare GCE, poly(PP)/GCE, CNT/GCE, CNT/poly(PP)/GCE, and AuNPs/CNT/poly(PP)/GCE were observed at 596 mV, 342 mV, 320 mV, 313 mV, and 27 mV, respectively. A shift in the overpotential to more negative direction and an enhancement in the peak current indicated that the AuNPs/CNT/poly(PP)/GC electrode presented an efficient electrocatalytic activity toward oxidation of hydrazine. Modified electrodes were characterized with High-resolution transmission electron microscopy (HRTEM), scanning electron microscopy (SEM), X-ray photoelectron spectroscopy (XPS) and electrochemical impedance spectroscopy (EIS). Amperometric current responses in the low hydrazine concentration range of 0.25–13 µM at the AuNPs/CNT/poly(PP)/GCE. The limit of detection (LOD) value was obtained to be 0.083 µM. A modified electrode was applied to naturel samples for hydrazine determination.

## 1. Introduction

Hydrazine is an inorganic molecule, colorless liquid and the simplest unique diamine molecule [1,2]. Hydrazine is also used in industrial, as a pesticide in agriculture, an intermediate compound in pharmaceutical, photographic chemical, a fuel in rocket systems,in the manufacture of metal films and as anodic depolarizers. It is known that hydrazine is a neurotoxin, carcinogenic and mutagenic subtance [3,4]. It can be absorbed through the skin and the acute toxicity of hydrazine affects central nervous system, our blood production, liver and kidney [5,6]. Hydrazine is classified as group B2 human carcinogens by World Health Organization (WHO) and Environmental Protection Agency (EPA) at a very low threshold value of 10 ppb (∼0.1 µM) [7]. The accurate and economical determination of hydrazine is important for analytical chemistry. Today, there are several methods for the determination of hydrazine such as chromatography [8], flow injection analysis with fluorimetric [9], amperometry [10], potentiometry [11], titrimetry [12] and chemiluminescence [13]. However, most of these methods require a complex process and at the same time the linear ranges between the hydrazine concentration and analytical signals are narrow [14,15]. Fortunately, electrochemical techniques offer the opportunity for portable, low cost, high sensitivity, selectivity, fast response and cost effective methodologies [16,17]. On the other hand the irreversible oxidation of hydrazine is kinetically slow and require high overpotential at carbon electrodes. Modified electrodes can be minimized hydrazine overpotential, and accelerated electron transfer rate for the detection of hydrazine [1,18,19].

Phenolphthalein is an available industrial product, an acid-base indicator in analytical chemistry and is obtained by synthesis of phenol and phthalic anhydride [20]. Phenolphthalein is colorless in both acidic and neutral medium but turns pink in basic solution.This is due to the structural change of the phenolphthalein molecule in the basic medium [21]. Phenolphthalein is not used in medical applications because it has some carcinogenic effects [22].

Metal nanoparticles have attracted considerable interest due to their unique properties such as large surface to volume ratio higher electronic and optical effects and catalytic properties as compared to the bulk equivalents [23]. It has been used many electrochemical fields due to mass transport, high catalytic activity and high effective surface area [24]. Metal nanoparticles such as palladium, gold, copper, platinum and silver have been found applications in many electrochemical fields [25].

Carbon nanotubes (CNTs) have large surface area, thermal stability, low electrical resistance, increased porosity and high electronic conductivity compared to the other carbon-based materials. They are used support materials to modify electrode surface and provide opportunities in sensor applications [26,27].

In the present study, we demonstrated a simple and versatile in situ approach for the fabrication of AuNPs/CNT/poly(PP)/GCE. Firstly, for this prurpose, GC electrode surface was covered with polyphenolphthalein [poly(PP)] by electropolymerization of phenolphthalein monomer using cyclic voltammetry. Then, carbon nanotube suspension was injected on the modified electrode surface. Finally, Au nanoparticles were electrodeposited on the CNT/poly(PP)/GCE surface. The prepared electrode was used for the determination of hydrazine at real samples. The modified electrode surfaces were characterized by XPS, EIS, HRTEM and SEM-EDX. The obtained modified electrode had good sensitivity, selectivity, and electrocatalytic activity for hydrazine.

## 2. Experimental

### 2.1. Chemicals and apparatus

Electrochemical measurements were carried out using Autolab 101 and 128N potentiostat electrochemical analyzer equipped with a three electrode system consisting of working electrode (glassy carbon electrode), an auxiliary platinum wire and an Ag/AgCl(sat.KCl) used as reference electrode. All electrochemical measurements performed at room temperature. Surface characterization of the modified electrodes were performed by using JEOL JEM 2100F high-resolution transmission electron microscopy (HRTEM) (JEOL Ltd., Tokyo, Japan), Thermo K-Alpha-monochromated high-performance XPS spectrometer (Thermo Fisher Scientific Inc.,Waltham, MA, USA) and Zeiss Gemini 500 SEM (Carl Zeiss Microscopy GmbH, Oberkochen, Germany).

Hydrazine was purchased from Aldrich Chemical Co. Inc. (Milwaukee, WI, USA) and phenolphthalein (PP) was obtained from Honeywell Riedel-de Haën GmbH (Seelze, Germany). Ethanol was used to prepare phenolphthalein solution. Multiwalled carbon nanotubes (CNT abbreviation used instead of MWCNT in electrode name) (purity>95% diameter 110–170 nm, length 9 µm) were purchased from Aldrich Chemical Co. Inc. Acetic acid (CH3COOH), boric acid (H_3_BO_3_) and phosphoric acid (H_3_PO_4_) (all 0.04  mol L^-1^) were used to preparing Britton–Robinson (BR) buffer solution. The pH of BR buffer solution was adjusted with 3 M NaOH solution. Distilled water was used to prepare all other solutions.

### 2.1. Modification of electrode

GCE was activated by polishing with different grade of Al_2_O_3_ slurry on a synthetic cloth and rinsed with ethanol and pure water. Electrochemical polymerization of PP was carried out in 0.1 M NaNO_3_ containing 1.0 mM PP solution by cyclic voltammetry. The polymerization voltammogram was obtained by repetitive 15 current-potential cycles from –1.2 V to +1.8 V. A 10 µL of multiwalled carbon nanotube suspension was injected on the poly(PP)/GCE surface and then the solvent evaporated by IR lamp exposure for 15 min. Finally, Au nano particles was deposited on the CNT/poly(PP)/GCE by cyclic voltammetry scanning between 0.60 V - -0.90 V in HAuCl_4_ with a scan rate of 100 mVs^-1^ for 10 cycles.

### 2.1. Sample analysis

The hydrazine content in Gediz and Karaçay Rivers waste water samples collected from industry in Manisa (Turkey) was investigated on the AuNPs/CNT/poly(PP)/GC electrode. Samples were prepared for analysis by first filtering through filter paper. The pH and conductivity of Gediz and Karaçay Rivers samples were measured to be pH 7.91, 1.46 mS and pH 7.82, 2.79 mS, respectively. 1 mL of water sample and 9 mL of pH 10.0 BR buffer solution was taken into voltammetric cell with a total volume of 10 mL. Then, determination of hydrazine was performed by using standard addition method with linear scanning voltammetry.

## 3. Results

### 3.1. Electropolymerization of PP and electrodeposited of gold nanoparticle

**Figure 1:**
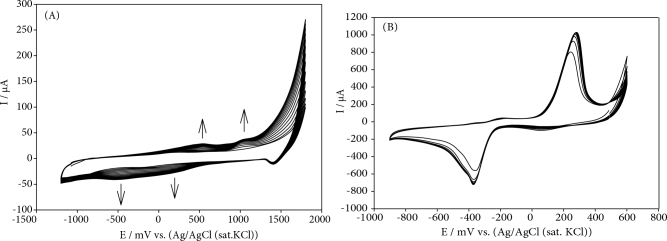
A) Cyclic voltammograms for electrochemical polymerization for 0.1 M NaNO3 containing 1.0 mM PP on bare GCE for 15 cycles, B) repetitive cyclic voltammograms recorded at 1.0 mM HAuCl_4_ solution 100 mVs^-1^ for 10 cycles.

The poly(PP) modified electrode was prepared by electrochemical polymerization of PP monomer on the GCE surface between –1.2 V and +1.8 V 200 mV s^-1^ in 0.1 M NaNO_3_ containing of 1.0 mM PP monomer (Figure 1A) [28]. During the electropolymerization process, in the anodic direction, two anodic peaks were observed at about +0.51 V and +1.04 V, while in the cathodic direction, two cathodic peaks were observed at about –0.5 V and +0.25 V. The observed anodic and cathodic peak currents were increased by consecutive current potential scan cycles number. In order to determine the most suitable polymerization electrolyte medium for phenolphthalein, the responses of the polymer film coated to the GCE surface in the presence of NaNO_3_ in different concentrations were investigated to 1 mM hydrazine (Figure S1). The optimum NaNO_3_ concentration was chosen as 0.1 M. The number of cycles and the monomer concentration can be controlled to adjust the thickness of the polymer. The optimum cycle number in PP electrochemical polymerization process was obtained as 15 cycles.

Figure 1B shows that the deposition of Au nanoparticles on the CNT/poly(PP)/GC electrode from 2.0 mM HAuCl_4_ subscripts solution (in 0.1 M HCl) by current potential scans from 0.60 V to –0.90 V for 10 cycles. The amount and size of Au nanoparticles deposited on the electrode surface, can be controlled by the electrodeposition process.

### 3.2. Surface characterization

**Figure 2:**
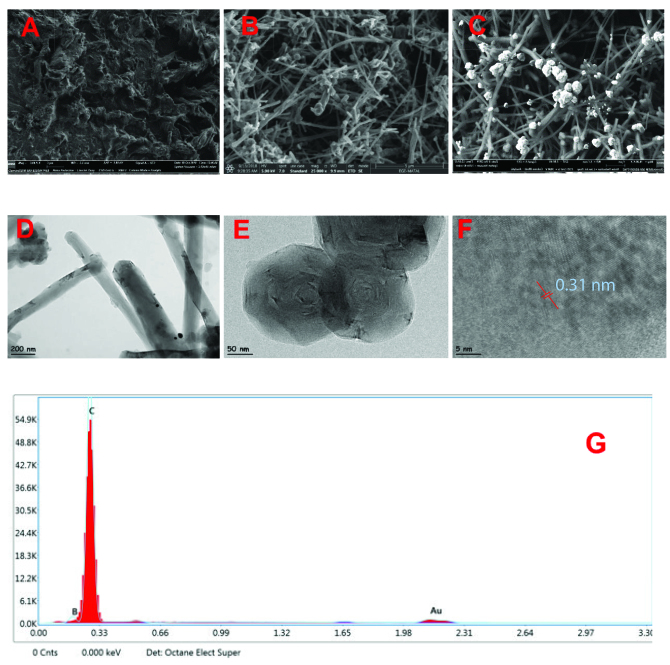
SEM images of modified electrodes, A) poly(PP)/GCE, B) poly(PP)/CNT/GCE, C) AuNPs/poly(PP)/CNT/GCE; HRTEM images of AuNPs/poly(PP)/CNT/GCE (D-F), EDX spectra of AuNPs/CNT/poly(PP)/GCE (G).

The morphology of poly(PP)/GCE (A), poly(PP)/CNT/GCE (B), AuNPs/CNT/poly(PP)/GCE (C) and (D) was examined by SEM, as shown in Figure 2. In Figure 2A, homogeneously distributed porous polymer surface was formed as a result of the polymerization of the phenolphthalein monomer on the bare electrode surface. The morphology of poly(PP)/CNT/GCE showed a network-like structure in Figure 2B. Nano Au particles were formed as bright, round-shaped which homogeneously distribution of Au nanoparticles on the modified electrode surface were seen in Figure 2C. The average particle size on AuNPs/CNT/poly(PP)/GCE was 200 nm. Au nanoparticles appear to be small spheres lined up on the CNT surface. Each of these spheres is composed of multiple Au particles and is lined up as stacking balls. Energy dispersive X-ray spectroscopy (EDX) study confirmed the presence Au nanoparticles decorating CNT/poly(PP)/GCE with the electrochemical deposition (Figure 2G).

The polymer and Au nanoparticles on AuNPs/poly(PP)/CNT/GCE were also examined by transmission electron microscopy. To obtained HRTEM images, the modified electrode surface was scraped and the obtained solid was taken into microcentrifuge tubes and analysed by adding 1 mL of high purity methanol. As shown in Figures 2D–2F, HRTEM images were taken at different magnification rates. It was demonstrated that poly(PP) structures formed as round spheres on CNT (Figures 2D and 2E). Au nanoparticles formed by combining as a ball on the modified electrode. The diameter of these ball-shaped Au nanoparticles was found to be 30 nm. At the same time, Au nanoparticles forming this sphere were presented in the form of agglomeration. The diameter of the Au nanoparticles in this agglomeration is about 3 nm. The distance between Au atom arrays in each Au nanoparticle was calculated to be 0.31 nm (Figure 2F).

When comparing SEM and TEM images, Au nanoparticle diameter was not the same due to the different preparation of SEM and TEM sample preparation procedures. In the SEM study, the electrochemical modified electrode surface can be accessed without any additional treatment. However, in the case of TEM operation, after the modified surface prepared electrochemically with both polymer and Au nanoparticles, the content must be scraped from the electrode surface with a sharp blade and transferred to the methanol solution and then to the TEM grids. Au nanoparticles can be released into small particles during TEM sample preparation steps compared to SEM conditions. Therefore, Au nanoparticle size in SEM images is higher than TEM images.

**Figure 3:**
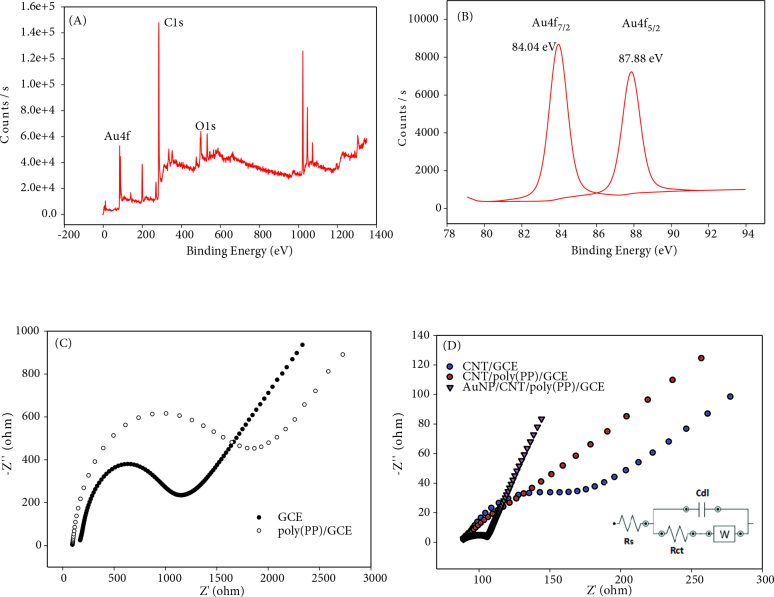
XPS spectra of AuNPs/CNT/poly(PP)/GCE, A) survey scan, B) binding energy of Au 4f_7/2_ and 4f_5/2_. Nyquist plots for modified electrode (C and D).

Characterizations and states of the components on the AuNPs/poly(PP)/CNT/GCE electrode surface were made using XPS spectra. Survey spectra recorded in a wide energy range, Figure 3A show the positions of the main characteristic peaks of the elements. These peaks are O1s, C1s, Au4f on the electrode surface (Figure 3B). The C signal, which has a binding energy of approximately 285 eV, originates from the polymer and corresponds to the core level of C1s [29]. The O signal having a binding energy of approximately 535 eV corresponds to the core level of O1s [30]. Two sharp peaks of Au4f were obtained (4f7/2 and 4f5/2) and their binding energies are 84.04 and 87.88 eV, respectively. These results should be attributed to the metallic gold form (Au0) [31,32].

Figures 3C and 3D indicate the results of electrochemical impedance spectroscopy on bare GCE and all modified electrodes in the presence of 5.0 mmolL^-1^ K_3_[Fe(CN)_6_]/K_4_[Fe(CN)_6_] containing 0.1 mol L^-1^ KCl solution at frequencies from 0.05 to 100.000 Hz. The Nyquist chart can consist of a semicircular section at higher frequencies and a linear section in the lower frequency range. In these EIS measurements, the charge transfer resistance (Rct) is calculated by fitting the R(R(CW)) circuit (Figure 3D inset). Figure 3C shows that poly(PP)/GCE had a large charge transfer resistance of about 1270 ohm (Table S1). The AuNPs/CNT/poly(PP)/GCE shows small semicircle diameter than of the CNT/GCE and CNT/poly(PP)/GCE. As a result, Au nanoparticles modified electrode has a lower interfacial charge transfer resistance.

### 3.3. Voltammetric behavior of hydrazine on bare and modified electrode

**Figure 4:**
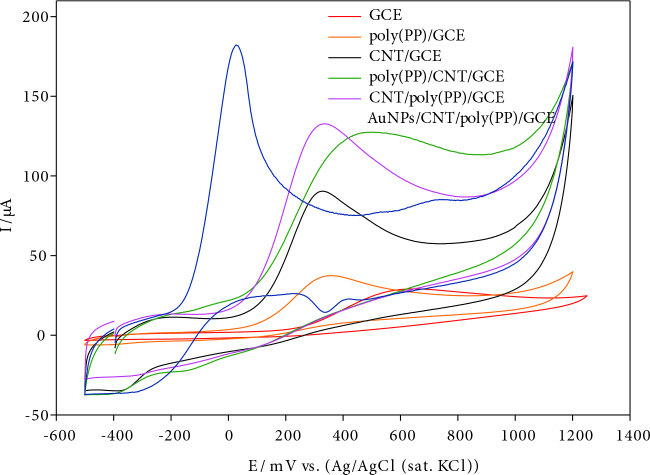
Cyclic voltamograms of 1.0 mM hydrazine on bare and modified electrode at scan rate of 5 0 mVs^-1^.

The electrochemical behaviour of hydrazine was investigated at the bare and modified electrodes by cyclic voltammetry. Figure 4 shows the cyclic voltammograms of bare GCE, poly(PP)/GCE, CNT/GCE, CNT/poly(PP)/GCE, AuNPs/CNT/poly(PP)/GCE in presence of 1.0 mM hydrazine in pH 10.0 BR buffer solution. An irreversible oxidation peak was appeared at 596 mV, 320 mV, 417 mV, 313 mV, and 27 mV at the bare GCE, CNT/GCE, poly(PP)/CNT/GCE, CNT/poly(PP)/GCE and AuNPs/CNT/poly(PP)/GCE, respectively. In terms of peak shape and location, the performance of AuNPs/CNT/poly(PP)/GCE towards hydrazine oxidation was found very effective compared to bare and other modified electrode.

Significant peak current enhancement about 8.79 times higher than bare GCE and 5.79 times higher than poly(PP)/GCE, 2.40 times higher than CNT/GCE and 2.36 times higher than poly(PP)/CNT/GCE and 1.64 times higher than CNT/poly(PP)/GCE were observed while the peak potential was shifted in negative direction, indicating a positive catalytic effect on hydrazine oxidation (Table S2). The improved peak characteristics indicate that faster electron transfer can be achieved by modifying Au metal nanoparticles on the CNT/poly(PP)/GCE surface.

### 3.4. Optimization studies of hydrazine oxidation at AuNPs/CNT/poly(PP)/GCE

**Figure 5:**
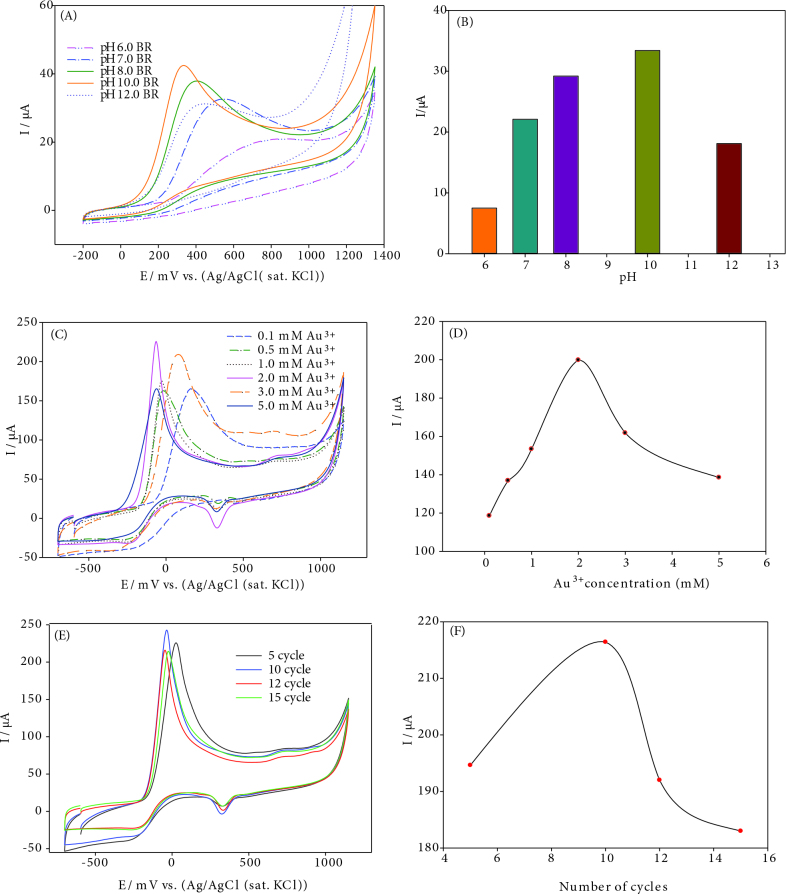
Cyclic voltammogram of 1.0 mM hydrazine in optimization parameters studies: A) the effect of BR buffer solution pH (6.0–12.0), B) pH-peak current graph, C) the effect of Au^3+^ concentrations, D) Au^3+^ concentrations-peak current, F) the effect of Au^3+^ cycle number on CNT/poly(PP)/GCE, G) Au^3+^ cycle number-peak current, scan rate: 50 mVs^-1^.

The thickness of the polymer film coated on the GCE and modified electrode surface strongly affects the oxidation behaviour of hydrazine. It can be easily controlled by changing the monomer concentration and repetitive cycle number during the electropolymerization procedure. The optimization graphics were shown in Figure 5. The effect of PP monomer concentration was investigated between 0.1 to 5.0 mmol L^-1^. A higher peak current for hydrazine oxidation peak was observed at AuNPs/CNT/poly(PP)/GCE, that polymer film was prepared in the presence of 1.0 mM concentration of PP monomer. In addition, the number of polymerization cycles was examined between 5 and 15 cycles to control the effect of hydrazine on peak flow. The best result for hydrazine oxidation was determined as 10 cycles.

The supporting electrolyte pH is significant parameter that can extremely change the analyte signal of the electrodes. To investigate the effect of write PH on the peak current and peak potential of hydrazine, the BR buffer in the pH 6.0–12.0 range at AuNPs/CNT/poly(PP)/GCE with cyclic voltammetry (Figures 5A and 5B). The highest current and low oxidation potential of hydrazine appeared in pH 10.0 BR buffer solution. For this reason, pH 10.0 BR buffer solution as supporting electrolyte was selected for further studies.

To investigate the catalytic effect of Au nanoparticles on hydrazine oxidation, the HAuCl_4_ solution concentration was changed in the range of 0.1–5.0 mM and cyclic voltammograms were recorded in the presence of pH 10.0 BR buffer (Figures 5C and 5D). As the Au^3+^ concentration range from 0.1 mM to 2.0 mM, the hydrazine peak potential shifted towards negative potentials while the increasing of the peak current of hydrazine. Therefore, 2.0 mM HAuCl_4_ concentration was chosen for preparation of Au nanoparticles in further studies.

As shown in Figures 5E and 5F, AuNPs/CNT/poly(PP)/GC electrode was prepared at different cycle numbers (5–15) from 1.0 mM HAuCl_4_ solution by cyclic voltammetry. The amount of nanoparticle was adjusted by changing the repeated cycle number in electrodeposition process. The maximum peak current of hydrazine was obtained at 10 cycle Au deposition and decreased thereafter. The optimum cycle number was chosen 10.

The scan rate dependence of cyclic voltammograms for the modified electrode in pH 10.0 BR buffer solution containing 1.0 mM hydrazine was used to get the information about the diffusion controlled current (Figure S2). As potential scan rate increased, the peak potential of hydrazine shifted to direction positive. The linearity of the current to the square root of the scan rate indicated that the current on the electrode surface was controlled by diffusion. Figure S2 demonstrates that the slope values of the plots are 0.82, 2.39, 3.27, 4.65, and 19.01 for GCE, CNT/GCE, poly(PP)/GCE, CNT/poly(PP)/GCE and AuNPs/CNT/poly(PP)/GCE, respectively.

### 3.5. The long-term stability

**Figure 6:**
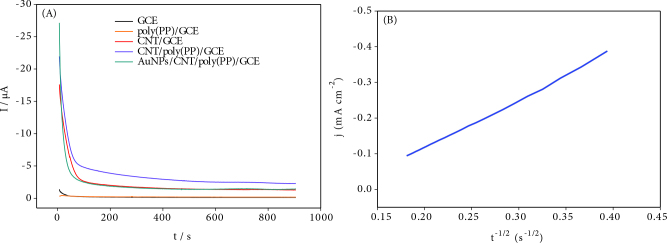
A) Chronoamperograms of the modified electrodes in pH 10.0BR buffer in the presence 1.0 mM hydrazine at the applied constant potential of –0.03 V, B) the plot of J vs. t^-1/2^ for AuNPs/CNT/write poly(FF)/GCE.

The performance of the five different electrodes toward the hydrazine oxidation reaction were tested by a chronoamperometry after a by holding –0.03 V constant potential at each electrode for 900 s. Figure 6A shows chronoamperograms for the bare GCE, poly(PP)/GCE, CNT/GCE, CNT/poly(PP)/GCE and AuNPs/CNT/poly(PP)/GCE in the presence 1.0 mM hydrazine. The current variation of bare GC and poly(PP)/GC modified electrodes was close to each other and current densities of them had lower than other modified electrodes. AuNPs/CNT/poly(PP)/GCE electrocatalyst had a better stability in 1.0 mM hydrazine compared to other electrodes.

The Cottrell equation can be used in chronoamperometric measurements to estimate the number of electrons (n) for oxidation of hydrazine in the modified electrode.

(1)IA=[nFACD1/2π1/2]t-1/2

where D is diffusion coefficients (cm^2^ s^-1^), C is the bulk hydrazine concentration (mol cm^-3^), A is the electrode area (0.0707 cm^2^), n: number of electrons. *F* is Faraday constant (96486 C moL^-1^).

The J (current density) versus t^-1/2^ graph can be plotted from the Cottrell equation and the current in the electrode press is linear under diffusion-controlled conditions. (Figure 6B and Table S3), If the diffusion coefficient for hydrazine is considered equal to 2.37 × 10^-5^ cm^2^ s^-1^[33], the number of electrons can be calculated from the Cottrell equation, which is in good agreement with values reported in literature [34]. The number of electrons was calculated as 4.2 for the oxidation of hydrazine at the AuNPs/CNT/poly(PP)/GCE. With the oxidation of hydrazine in basic medium, nitrogen, water and four-electron are formed in the products as follows:

N2H4+4OH-→N2+4H2O+4e-

To test the intraday repeatability of the modified electrode, five different modified electrodes were prepared by the same way and their responses toward the 1.0 mM hydrazine at pH 10.0 BR buffer were investigated by cyclic voltammetry (Figure S3). The relative standard deviation (RSD) (n = 5) value of the hydrazine peak current was found to be 7.75% at the AuNPs/CNT/poly(PP)/GC electrode. The current change was plotted by measuring the peak current of 1.0 mM hydrazine on different days for 8 days in pH 10.0 BR buffer at AuNPs/CNT/poly(PP)/GCE.

### 3.6. Determination of hydrazine

**Figure 7:**
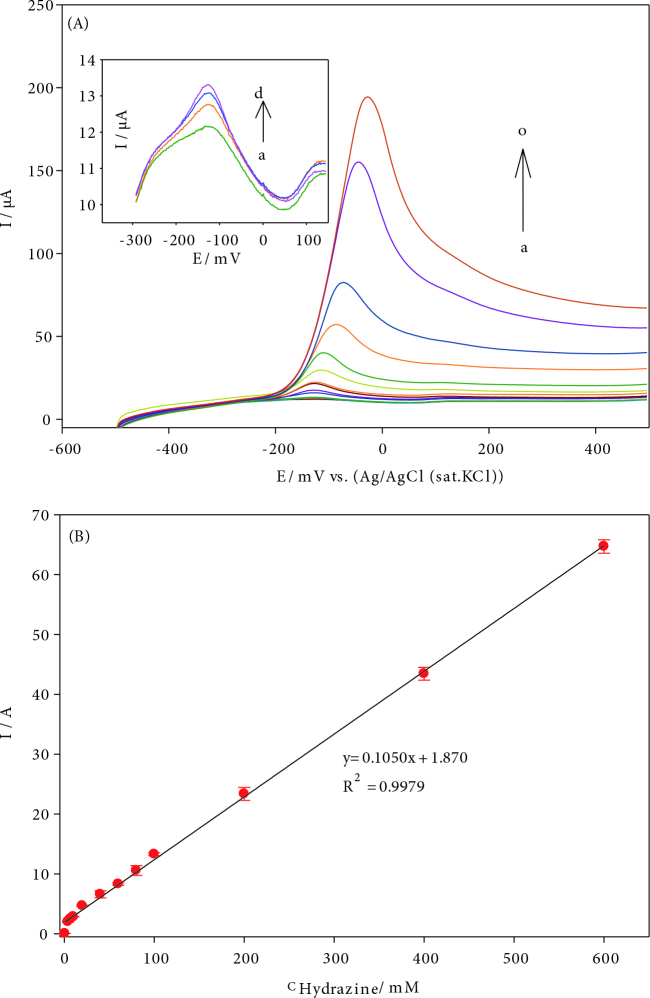
A) Linear sweep voltammograms of hydrazine concentrations, (a–o): 4–1000 μM hydrazine, B) the calibration curves for hydrazine at AuNPs/CNT/poly(PP)/GCE.

LSV method was used for hydrazine determination at AuNPs/CNT/poly(PP)/GC electrode. Figure 7A shows that hydrazine oxidation peak was at applied potential -0.03 V. As the hydrazine concentrations were added to the pH 10.0BR buffer solution, the peak current of the hydrazine increased. As shown in Figure 7B, calibration graph was obtained with the peak current values in response to hydrazine concentrations in the range of 4–1000 µM. The linear regression equation is: i(μA) = 0.102 C(μM) +2.376, (R^2^: 0.9965). The limit of detection (LOD) was found to be 1.33 µM for AuNPs/CNT/poly(PP)/GC electrode.

**Figure 8:**
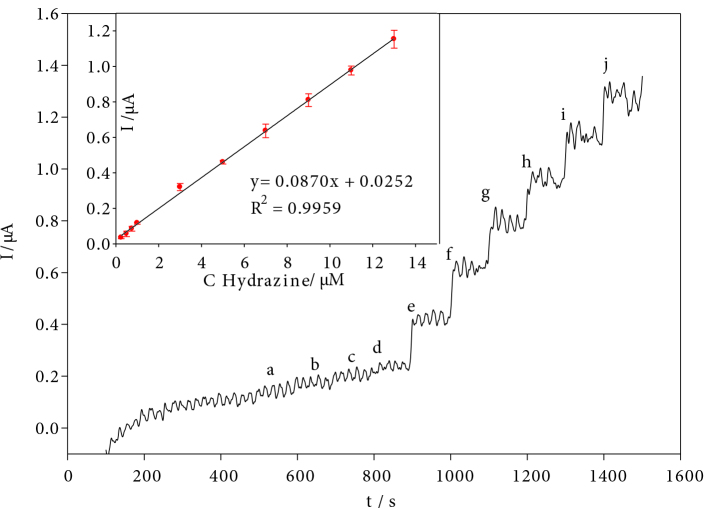
Amperograms of the AuNPs/CNT/poly(PP)/GCE in pH 10.0 BR buffer, applied potential –0.03 V. (a) 0.25, (b) 0.5, (c) 0.75, (d) 1, (e) 3, (f) 5, (g) 7, (h) 9, (i) 11, and (j) 13 μM hydrazine. (Inset: the calibration graph).

Amperometric responses of the hydrazine in the equal concentration range were compared to the GCE, CNT/GCE and AuNPs/CNT/poly(PP)/GCE in the Figure S4. Since the highest slope is AuNPs/CNT/poly(PP)/GCE, it has been used in chronoamperometric studies. For amperometric determination, hydrazine was added at increasing concentrations into stirred pH 10.0 BR buffer solution at an applied potential at –0.03 V and the amperometric current-time (i-t) graph was obtained. Figure 8 shows amperometric current responses in the low hydrazine concentration range of 0.25–13 µM at the AuNPs/CNT/poly(PP)/GCE. With each hydrazine addition, the current gradually increased. Since it reached saturation after a certain time, the peak current remained constant. When the peak current versus the hydrazine concentration was plotted, a linear calibration graph was obtained. The linear regression equation is: I(μA) = 0.0874 C(µM) + 0.0315, (R2: 0.9975). The limit of detection (LOD) was calculated to be 0.083 µM (at a signal-to-noise ratio of 3). The obtained results show that the chronoamperometric method determined lower hydrazine concentration. The LOD value obtained for hydrazine is 0.083 µM, it offers the opportunity to be determined below the WHO and EPA threshold value of 10 ppb (∼0.1 µM). The previous studies on the determination of hydrazine are given for comparison in Table 1.

**Table 1:** Comparison of several hydrazine sensors at different modified electrodes.

Electrode	Method	LOD (µM)	Linear range (µM)	Ref.
Ag-nanozeolite/CPE	Amperometry	3.72	12–15000	35
Ag/PPy/GCE	CV	0.2	0.5–1000 and 1000–10000	36
Pd decorated bamboo MWCNTs	LSV	10	56–157	37
ZrHCF/AuPtNPs/NFs/GCE	Amperometry	0.09	0.15–112.5	38
AuNPs/poly(L-serine)/GCE	LSV	0.5	1–1000	39
AuNPs/CNT/poly(PP)/GCE	LSV Amperometry	1.33 0.083	4–1000 0.25–13	This work

### 3.7. Interference effect on hydrazine determination

**Figure 9:**
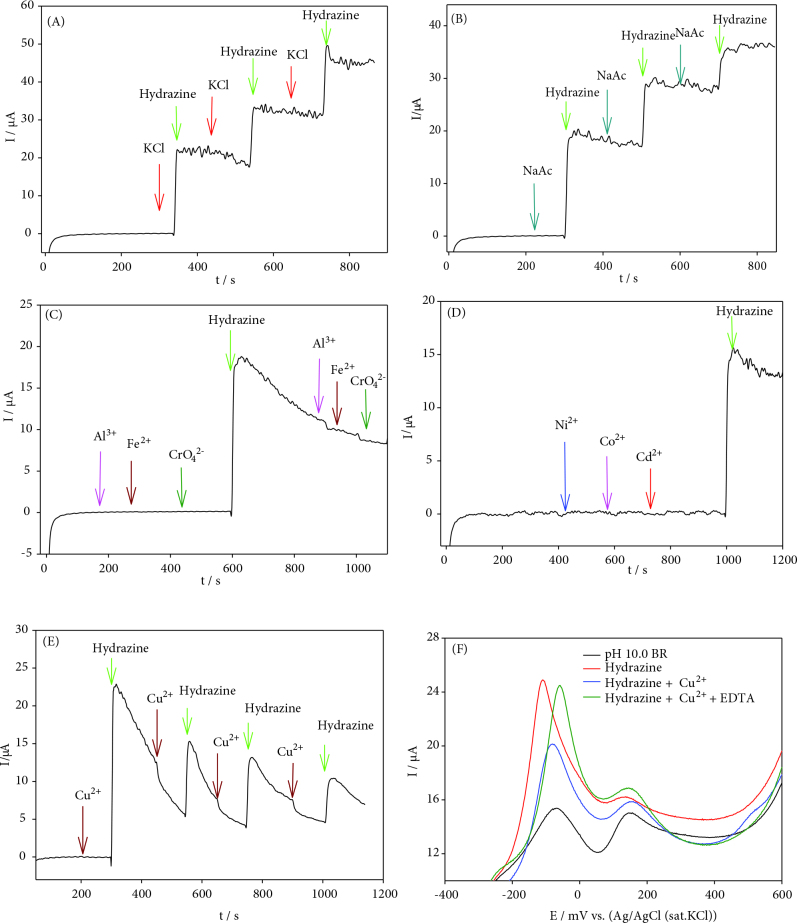
Interference effect on determination of hydrazine at AuNPs/CNT/poly(PP)GC electrode A) KCl, B) sodium acetate (NaAc), C) AlP3^+^P, FeP2^+^P, CrO_4_P2^-^P, D) NiP2^+^P, CoP2^+^P, CdP2^+^P, E) elimination of CuP2^+^Pwith EDTA.

Interferences such as K^+^, Cd^2+^, Ni^2+^, Co^2+^, Al^3+^, Fe^2+^, Cu^2+^, Cl^-^, NO_3_^-^, SO_4_^2-^, CrO_4_^4-^, and sodium acetate for the determination of hydrazine were investigated using amperometry technique at AuNPs/CNT/poly(PP)/GC electrode (Figure 9). The amperograms showed that 50 times excess of sodium acetate, K^+^, Cd^2+^, Ni^2+^, Co^2+^, Al^3+^, Fe^2+^ Cl^-^, NO_3_^-^, SO_4_^2-^, CrO_4_^2-^ had no interference on determination of hydrazine. When Cu^2+^ was added to the pH 10.0 BR buffer, the hydrazine peak current decreased and the precipitation occurred due to the basic medium at high concentrations. As a result, Cu^2+^ showed significant interference in the determination of hydrazine. To eliminated Cu^2+^ interference in the hydrazine signal, EDTA was added to the same medium and the linear sweep voltammograms were recorded (Figure 9F). After the addition of EDTA, the peak current signal of hydrazine almost returned to its initial state.

### 3.8. Real sample analysis

To investigate for the determination of hydrazine in real samples, Gediz and Karaçay Rivers water were analyzed at the AuNPs/CNT/poly(PP)/GC electrode by amperometry. 1.0 mL of water sample and 9.0 mL of pH 10.0 BR buffer solution was taken into voltammetric cell with a total volume of 10.0 mL. Then, hydrazine determination was made by using standard addition method with linear sweep voltammetry. No hydrazine was detected in water samples taken from Karaçay and Gediz Rivers. For AuNPs/CNT/poly(PP)/GC electrode, recovery studies were investigated for Karaçay and Gediz Rivers samples for 2 different concentrations (50 and 500 μM). The results are given in Table 2.

**Table 2:** Recovery analysis of hydrazine determination in Karaçay and Gediz Rivers samples at the modified electrode (n = 3).

Sample	Spiked (µM)	Founded (µM)	Recovery (%)	R.S.D. (%)
Karaçay River	0 50 500	- 55 540	- 110 108	- 4.1 3.4
Gediz River	0 50 500	- 56 516	- 112 103	- 4.2 0.8

## 4. Conclusion

In this study, we have developed a novel modified electrode for hydrazine determination. The AuNPs/CNT/poly(PP)/GCE was prepared electrochemically and this modified electrode demonstrated electrocatalytic activity towards the hydrazine oxidation in pH 10.0 BR buffer solution. The Au nanoparticle modified CNT/poly(PP)/GC electrode has shown a better stability, sensitivity and selectivity than the other electrodes. Since fabricated electrode negatively shifts the oxidation potential of hydrazine, it is also very promising for use as an anode electrode in direct hydrazine fuel cells.

Supplementary Materials
