# Supplementary material for: Sensitive determination of hydrazine using poly(phenolphthalein), Au nanoparticles and multiwalled carbon nanotubes modified glassy carbon electrode
Source: Turk J Chem. 2021 Feb 17;45(1):167–80. doi: 10.3906/kim-2009-12 (PMC7925322; doi:10.3906/kim-2009-12)
Supplement: Supplementary file 1 — Supplementary Materials [file turkjchem-45-167-sup001.pdf]

## Supporting information

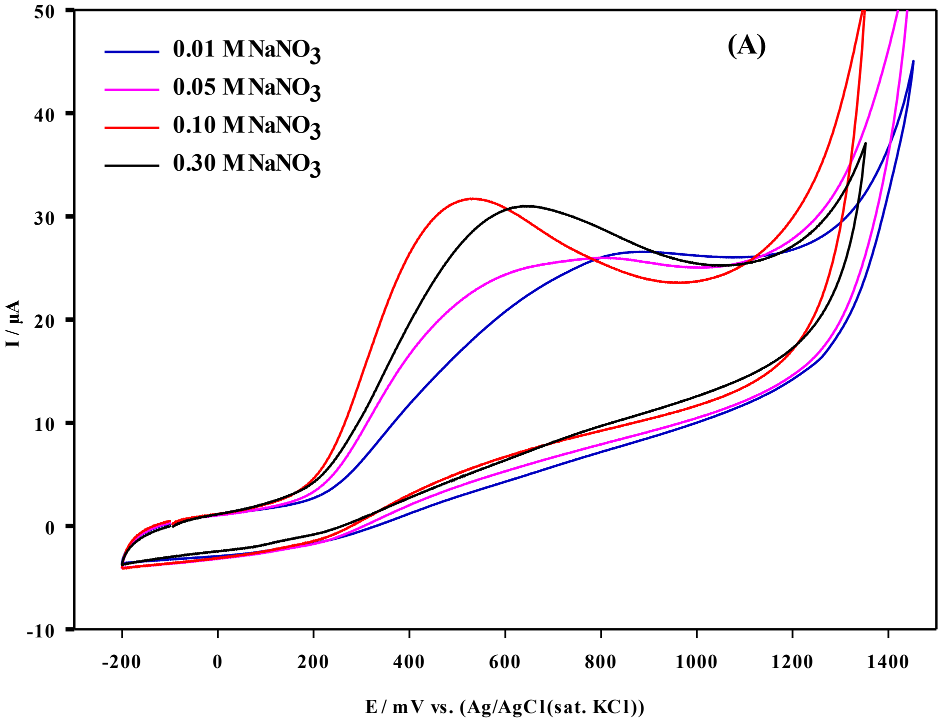


**Figure S1.** Cyclic voltammogram of hydrazine at the electrode obtained from phenolphthalein polymerization at different $NaNO_3$ concentrations.

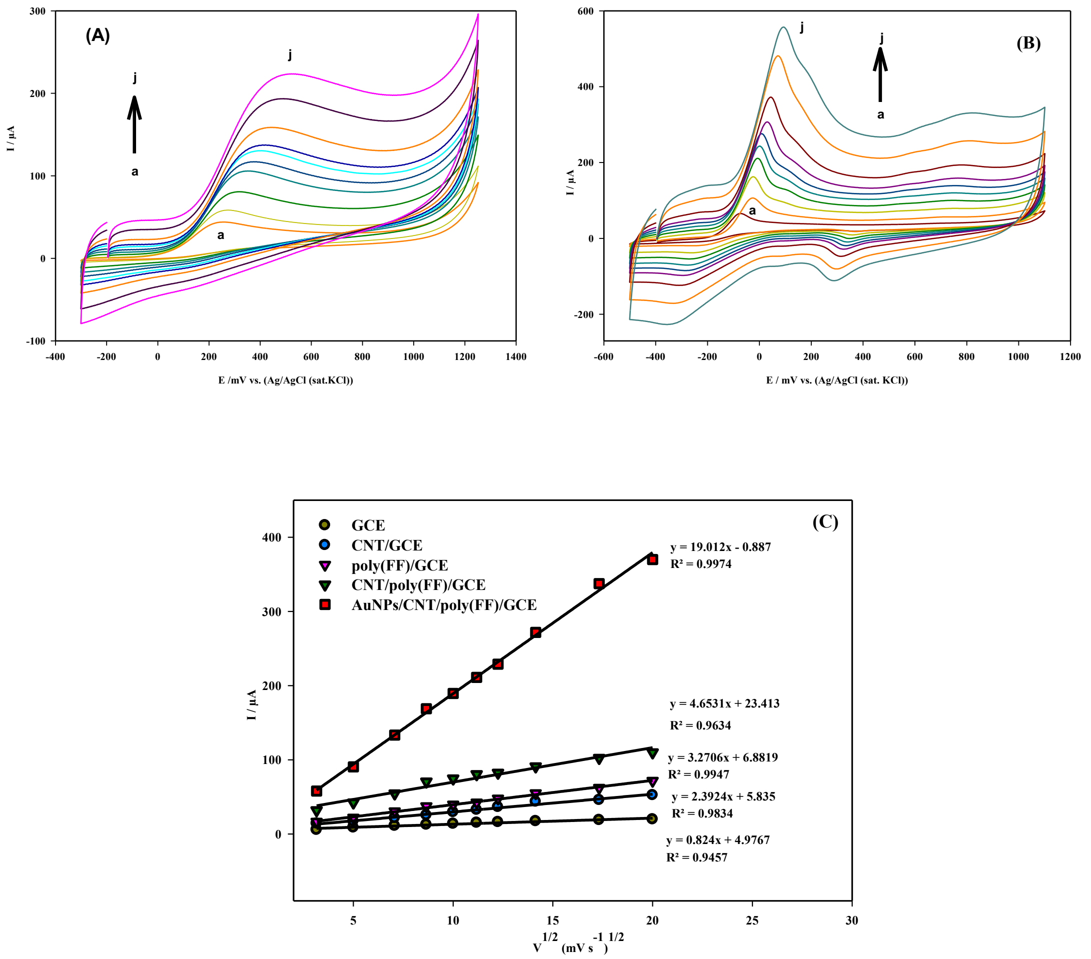


**Figure S2.** Cyclic voltammograms of hydrazine at different scan rates A) CNT/poly(PP)/GCE, B) AuNPs/CNT/poly(PP)/GCE, C) the plot of $I_{pa}$ vs. $v^{1/2}$ for the hydrazine oxidation at different electrodes a) 10, b) 25, c) 50, d) 75, e) 100, f) 125, g) 150, h) 200, i) 300, j) 400 mVs$^{-1}$.

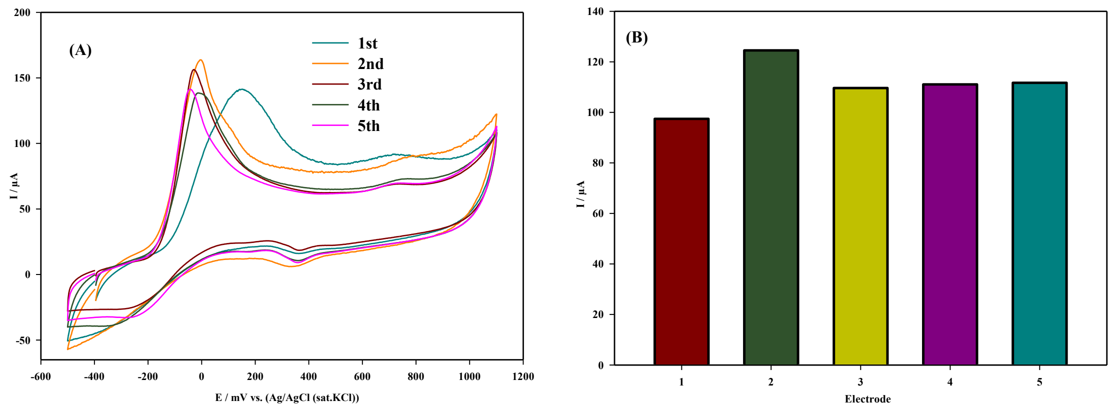


**Figure S3.** Intraday measurements for 1.0 mM hydrazine in AuNPs/CNT/poly(PP)/GC electrode, A) cyclic voltammograms, B) the graph of the response electrode.

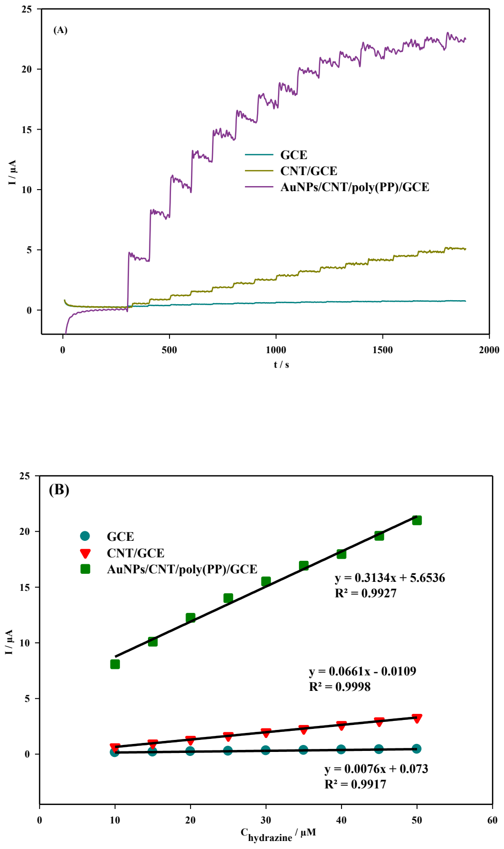


**Figure S4.** Amperometric hydrazine determination in pH 10.0 BR buffer at GC, CNT/GC and AuNPs/CNT/poly(PP)/GC electrodes A) chronoamperomogram, B) calibration graph.

**Table S1.** Resistance values obtained from different modified electrodes.

| Electrodes | Rct (ohm) |
|---|---|
| GCE | 844.0 |
| Poly(PP)/GCE | 1270.0 |
| CNT/GCE | 77.0 |
| CNT/poly(PP)/GCE | 37.3 |
| AuNPs/CNT/poly(PP)/GCE | 8.4 |

**Table S2.** The oxidation peak current and potential values of hydrazine at the modified electrodes.

| **Electrode surfaces** | **E/mV** | **I/µA** | **ΔE/(mV)** | **ΔI/(µA)** |
|---|---|---|---|---|
| **GCE** | 596 | 17.0 | - | - |
| **Poly(PP)/GCE** | 342 | 25.8 | 254 | 8.7 |
| **CNT/GCE** | 320 | 62.4 | 276 | 45.3 |
| **Poly(PP)/CNT/GCE** | 417 | 63.4 | 179 | 46.3 |
| **CNT/poly(PP)/GCE** | 313 | 91.2 | 283 | 74.1 |
| **AuNPs/CNT/poly(PP)/GCE** | 27 | 149.5 | 569 | 132.4 |

**Table S3.** Cottrel graphic parameters for hydrazine at the AuNP/CNT/write poly(FF)/GCE.

| Cottrel parameters | Values |
|---|---|
| The slope | –1.1557 |
| intercept | 0.1151 |
| RP2 | 0.9994 |
| n | 4.2 |
